# Impact of Multidrug-Resistant Uropathogens on Mortality in Elderly Patients with Urinary Tract Infections: A Multicenter Retrospective Study

**DOI:** 10.3390/diagnostics16111708

**Published:** 2026-06-02

**Authors:** Pınar Yürük Atasoy, Sevil Alkan, Dilek Bulut, Pelin Beyza Ünal, Derya Seyman, Ayşegül Seremet Keskin, Ayşegül Tuna, Ahmet Şahin, Mustafa Serhat Şahinoğlu, Şafak Kaya, Yasemin Çağ, Nurşen Demirkol Kaya, Mehmet Çelik, Deniz Gür Altunay, Cumhur Artuk, Bircan Kayaaslan

**Affiliations:** 1 Department of Infectious Diseases and Clinical Microbiology, Ankara Bilkent City Hospital, Ankara 06502, Turkey; 2 Department of Infectious Diseases and Clinical Microbiology, Faculty of Medicine, Canakkale Onsekiz Mart University, Canakkale 17100, Turkey; s-ewil@hotmail.com; 3 Department of Infectious Diseases and Clinical Microbiology, Ankara Etlik City Hospital, Ankara 06010, Turkey; dilekerdim@hotmail.com (D.B.); pelinbeyza11@gmail.com (P.B.Ü.); 4 Department of Infectious Diseases and Clinical Microbiology, Antalya Training and Research Hospital, University of Health Sciences, Antalya 07050, Turkey; seymander@gmail.com (D.S.); aseremetkeskin@yahoo.com (A.S.K.); 5 Department of Infectious Diseases and Clinical Microbiology, Faculty of Medicine, Kirikkale University, Kirikkale 71450, Turkey; draaslan87@gmail.com; 6 Department of Infectious Diseases and Clinical Microbiology, Dr. Ersin Arslan Training and Research Hospital, Gaziantep Islamic Science and Technology University, Gaziantep 27010, Turkey; ahmet27sahin@hotmail.com; 7 Department of Infectious Diseases and Clinical Microbiology, Faculty of Medicine, Mersin University, Mersin 33343, Turkey; drserhatsahinoglu@gmail.com; 8 Department of Infectious Diseases and Clinical Microbiology, Gazi Yasargil Training and Research Hospital, University of Health Sciences, Diyarbakir 21090, Turkey; ksafak76@gmail.com; 9 Department of Infectious Diseases and Clinical Microbiology, Goztepe Prof. Dr. Suleyman Yalcin City Hospital, Faculty of Medicine, Istanbul Medeniyet University, Istanbul 34722, Turkey; dryasemincag@gmail.com (Y.Ç.); nursendmrkl@hotmail.com (N.D.K.); 10 Department of Infectious Diseases and Clinical Microbiology, Faculty of Medicine, Harran University, Sanliurfa 63300, Turkey; dr.mcelik12@gmail.com; 11 Department of Infectious Diseases and Clinical Microbiology, Kocaeli City Hospital, Kocaeli 41050, Turkey; gur.deniz@hotmail.com; 12 Department of Infectious Diseases and Clinical Microbiology, Gulhane Training and Research Hospital, University of Health Sciences, Ankara 06010, Turkey; cumhur.artuk@sbu.edu.tr; 13 Department of Infectious Diseases and Clinical Microbiology, Ankara City Hospital, Ankara Yildirim Beyazit University, Ankara 06800, Turkey; bkayaaslan@aybu.edu.tr

**Keywords:** elderly patients, urinary tract infection, emergency department, mortality, multidrug-resistant bacteria

## Abstract

**Background/Objectives:** Urinary tract infections (UTIs) in older adults are common and potentially life-threatening conditions that often present with atypical symptoms. Early identification of prognostic factors is essential to improve clinical outcomes and reduce mortality in this vulnerable population. **Methods:** This retrospective, multicenter study included patients aged ≥65 years who were hospitalized with a diagnosis of UTI between January 2019 and December 2023. Diagnoses were established by infectious disease specialists based on clinical findings and microbiological confirmation in accordance with international guidelines. Only patients with urine cultures showing ≥100,000 CFU/mL bacterial growth were included. Demographic, clinical, laboratory, and microbiological data were analyzed. Multivariable logistic regression was used to identify independent predictors of in-hospital mortality. **Results:** A total of 1175 patients (median age: 75 years; 51.1% male) were included. The in-hospital mortality rate was 14.6%, and 25.6% required intensive care unit (ICU) admission. Multidrug-resistant (MDR) bacteria were detected in 63.6% of isolates, and bacteremia was present in 24.3% of cases. In multivariable analysis, MDR/ESBL positivity (OR: 2.09, 95% CI: 1.24–3.50, *p* = 0.005), bacteremia (OR: 2.10, 95% CI: 1.32–3.35, *p* = 0.002), and SOFA score (OR: 1.53, 95% CI: 1.42–1.65, *p* < 0.001) were independently associated with in-hospital mortality. Age and altered mental status were also significant predictors, while CRP and procalcitonin lost significance after adjustment. **Conclusions:** UTIs in elderly patients are associated with substantial morbidity and mortality. Multidrug-resistant pathogens, bacteremia, and disease severity play a central role in determining outcomes. Early identification of high-risk patients using clinical severity scores and microbiological data may improve risk stratification and guide timely, targeted therapeutic interventions.

## 1. Introduction

Elderly patients experience infections more frequently and with greater severity due to immunosenescence, comorbidities, and overall health decline [1]. Factors such as renal impairment, bladder dysfunction, urinary retention, catheter use, prostate disease, vaginal flora changes, hospitalization, and long-term care residence increase the prevalence of urinary tract infections (UTIs) [2]. Unlike younger adults, who typically present with fever, flank pain, and dysuria, elderly patients often exhibit nonspecific symptoms, such as confusion, fatigue, appetite loss, or altered consciousness, leading to delayed diagnosis and higher risks of bacteremia, sepsis, and uroseptic shock [3,4].

UTIs are a major cause of emergency department visits in older adults, representing a leading source of community-acquired bacteremia and the second most common infection-related hospitalization after pneumonia [5,6,7,8,9]. Their atypical presentation frequently results in underdiagnosis, as highlighted in both practice and research [10].

This study aimed to assess the clinical features, complicating factors, isolated pathogens, and mortality risk factors in elderly patients admitted to the emergency department with UTIs.

We hypothesized that multidrug-resistant uropathogens and bacteremia are major determinants of poor clinical outcomes, including intensive care unit admission and mortality, in elderly patients with urinary tract infections.

## 2. Materials and Methods

### 2.1. Study Design and Setting

This retrospective, multicenter study was conducted between January 2019 and December 2023 and included patients aged ≥65 years who presented to the emergency department of 12 centers. Only patients evaluated and diagnosed with urinary tract infections (UTIs) by infectious disease specialists during hospitalization were included.

### 2.2. Definition of UTI

The diagnosis of UTI was based on the presence of urinary or systemic symptoms (such as fever, dysuria, impaired oral intake, or altered mental status) together with microbiological confirmation (≥10^5^ CFU/mL bacterial growth in urine culture), in accordance with international guidelines (EAU/IDSA). In addition to urinary tract infections, related urological conditions such as prostatitis were also included when diagnosed by the treating physician based on compatible clinical findings, microbiological results, and available physical examination and/or imaging findings documented in the medical records.

### 2.3. Exclusion Criteria

Patients with asymptomatic bacteriuria, isolated cystitis, or a clear extra-urinary source of infection at admission were excluded.

### 2.4. Data Collection and Measurements

Demographic data, comorbidities, clinical findings, and laboratory parameters were recorded. qSOFA and Charlson Comorbidity Index scores were calculated. Microbiological analyses included identification of pathogens, ESBL production, and multidrug resistance (MDR). Bacteremia was defined as the isolation of the same pathogen in blood cultures. Treatment modification was defined as a change in the empirical antibiotic regimen according to urine culture and antimicrobial susceptibility results, including escalation to broader-spectrum therapy or de-escalation to narrower-spectrum agents when appropriate. The primary outcome of the study was in-hospital mortality. Although 14-day and 28-day mortality data were available, the primary analysis was based on overall in-hospital mortality and these time-specific outcomes were not analyzed separately.

### 2.5. Statistical Analysis

Categorical variables were compared using the chi-square test or Fisher’s exact test, as appropriate. Continuous variables were analyzed using the independent-samples *t*-test or Mann–Whitney U test, depending on data distribution.

Variables with clinical relevance or *p* < 0.10 in univariate analysis were entered into the multivariable logistic regression model. In the revised analysis, the multivariable model was reconstructed to include key clinical severity markers and microbiological factors. MDR/ESBL positivity was considered the main exposure variable. Clinical severity indicators, including SOFA score, bacteremia, C-reactive protein (CRP), and procalcitonin, were incorporated into the model. CRP and procalcitonin values were log-transformed due to their non-normal distribution.

Adjusted odds ratios (ORs) with 95% confidence intervals (CIs) were calculated. Multicollinearity was assessed using variance inflation factors (VIF), with values <5 considered acceptable.

To assess the robustness of the findings, a sensitivity analysis was performed using 28-day mortality as an alternative outcome. Detailed results of the sensitivity and stratified analyses are provided in Supplementary Tables S1–S3. ROC curves for all evaluated outcomes are presented in Figure S1.

A two-sided *p* value < 0.05 was considered statistically significant. Statistical analyses were performed using SPSS version 26.0 (IBM Corp., Armonk, NY, USA).

## 3. Results

A total of 1175 patients diagnosed with UTI in the emergency department were included in this study. The median age was 75 years (range: 65–100), with 48.9% females (*n* = 574) and 51.1% males (*n* = 601). Long-term care facility residents accounted for 10.5% of the total. Community-acquired infections comprised 67.3% of the cases, whereas 28.9% (*n* = 340) were healthcare-associated.

The most common complicating factors for UTI, in order of frequency, were antibiotic use in the last three months (45.6%), recurrent urinary tract infection (35.7%), and hospitalization within the last 30 days (29.9%). At least one urinary symptom was detected in 77.1% (*n* = 906) of the patients. The most frequently reported urinary symptoms were dysuria (61.9%), urgency (27.2%), pollakiuria (22.4%), urinary incontinence (20.4%), and hematuria (11.8%). Systemic symptoms were observed in 98% (*n* = 1152) of the patients, and the most common symptoms were fever (65.7%), impaired oral intake (48.2%), vomiting (32.7%), nausea (31.4%), flank pain (30.8%), mental status changes (30.6%), chills (28.4%), and abdominal pain (24.4%).

At admission, leukocytosis was observed in 758 patients (64.5%). Mean laboratory values were: procalcitonin (*n* = 967) 8.19 ± 16.3 µg/L, creatinine (*n* = 1148) 3.16 ± 17.2 mg/dL, BUN (*n* = 1169) 59.6 ± 62.5 mg/dL, and lactate (*n* = 947) 2.2 ± 1.9 mmol/L. The median Charlson Comorbidity Index was 5 (range, 0–90), and the median SOFA score was 2 (range, 0–14).

Urine cultures most commonly identified *Escherichia coli* (*E. coli*) (57.3%) and *Klebsiella pneumoniae* (*K. pneumoniae*) (22.8%), followed by other Gram-negative bacteria (12.1%) and Enterococcus spp. (4.3%), and Staphylococcus aureus (0.8%). ESBL production was observed in 60.3% of *E. coli* and 48.1% of *K. pneumoniae* isolates, whereas MDR bacteria were present in 63.6% of all isolates. Bacteremia was detected in 24.3% of patients with blood cultures, most commonly *E. coli* (12.7%). Among patients with the same microorganism in both urine and blood cultures, the distributions were as follows: *E. coli* (12.2%), *K. pneumoniae* (5.8%), other Gram-negative pathogens (2.6%), *Enterococcus* spp. (0.9%), and *Staphylococcus aureus* (0.4%).

Empirical antibiotics were initiated in 99.6% of patients, primarily carbapenems (39.7%), cephalosporins (33%), and piperacillin-tazobactam (13.4%). Regimen modifications based on urine culture antibiograms were made in 50.3% (*n* = 591) of the cases, with 54.9% (*n* = 325) requiring broad-spectrum escalation.

After treatment modification according to culture and antimicrobial susceptibility results, 26.9% of these patients required intensive care, and 16.2% had in-hospital mortality. The mean duration of antibiotic therapy was 11.3 ± 3.9 days.

Of the 856 patients (72.8%) with follow-up urine cultures, 67.7% (*n* = 796) achieved a microbiological cure, showing no bacterial growth post-treatment. However, in 69 patients (5.9%), bacterial growth persisted despite effective therapy, indicating a failure of source control.

A total of 85.3% (*n* = 1003) of patients were discharged, 25.6% (*n* = 301) required intensive care, and 14.6% (*n* = 172) experienced in-hospital mortality. Although 14-day and 28-day mortality data were available, the primary analysis was based on in-hospital mortality.

Patients were grouped according to ICU admission and mortality. ICU-admitted patients were older (78 vs. 74 years, *p* < 0.001), with no gender difference (*p* = 0.692). ICU admission was associated with chronic kidney disease, urolithiasis, urinary catheter use, neurogenic bladder, immunosuppression, and prostatitis (*p* ≤ 0.007). In mortality cases (*n* = 172), median age was higher (78 vs. 75 years, *p* = 0.001), with no gender difference (*p* = 0.623). Mortality was associated with chronic kidney disease, urolithiasis, neurogenic bladder, immunosuppression, renal tumors, prostatitis, and non-urinary comorbidities (*p* ≤ 0.022). Charlson scores showed no significant difference (*p* = 0.891) (Table 1).

Comprehensive univariate analyses for in-hospital mortality and ICU admission, including odds ratios with 95% confidence intervals, are presented in Table 2 and Table 3.

The results of the revised multivariable logistic regression analysis for in-hospital mortality are presented in Table 4. In this model, MDR/ESBL positivity was identified as an independent predictor of mortality, associated with approximately a two-fold increase in risk (OR: 2.09, 95% CI: 1.24–3.50, *p* = 0.005). Bacteremia was also independently associated with mortality (OR: 2.10, 95% CI: 1.32–3.35, *p* = 0.002). The SOFA score emerged as the strongest predictor, with each unit increase associated with a significant increase in mortality risk (OR: 1.53, 95% CI: 1.42–1.65, *p* < 0.001). Other variables, including CRP, procalcitonin, Charlson comorbidity index, and treatment modification, were not independently associated with mortality after adjustment. Mental status changes and age showed modest but significant associations. The direction of all associations in the revised model was consistent with clinical expectations. The model demonstrated good discriminative performance (AUC = 0.858), and no significant multicollinearity was detected, with all VIF values < 5.

Similar findings were observed in the 28-day mortality analysis, where MDR/ESBL positivity, bacteremia, and SOFA score remained significant predictors, confirming the robustness of the model (Table 5).

Stratified analyses by sex and catheter status showed consistent results and are presented in Supplementary Tables S1 and S2.

In ICU-admitted patients, CRP, procalcitonin, and SOFA scores were significantly higher, while Charlson scores showed no significant difference. Similarly, in patients with in-hospital mortality, CRP, procalcitonin, and SOFA scores were significantly elevated, whereas Charlson scores did not differ significantly.

*E. coli* and *K. pneumoniae* in urine cultures were significantly associated with both ICU admission and mortality. Among *E. coli* cases, ICU admission rates were higher in ESBL and MDR strains. *K. pneumoniae* cases showed higher ICU admission and mortality rates, particularly in MDR strains. In bacteremia cases, both ICU admission and in-hospital mortality rates were markedly higher.

## 4. Discussion

Infections are more frequent in the elderly than in young adults and often necessitate hospitalization. The literature indicates that over half of infection-related deaths occur in hospitals, with a significant proportion in the elderly. In our study, the median patient age was 75 years (range: 65–100), aligning with reports that UTIs requiring hospitalization are most common in the 75–84 age group [1].

The diagnosis of UTI in the elderly is challenging because of asymptomatic bacteriuria and nonspecific symptoms [11]. The lack of typical infection signs and the influence of comorbidities further complicate the diagnosis, especially in emergency settings [12]. To address this, our study included only patients with classical urinary symptoms, systemic or non-specific clinical findings (e.g., general health deterioration, reduced oral intake, and abdominal pain), and documented bacterial growth in urine cultures.

In our study, *E. coli* (57.3%) and *K. pneumoniae* (22.8%) were the most frequently isolated pathogens, consistent with previous reports [2,13]. The ESBL rates were 60.3% in *E. coli* and 48.1% in *K. pneumoniae* isolates. Studies conducted in our country have reported an increasing trend in these rates over the years [14,15,16,17]. This trend has led to the frequent use of broad-spectrum antibiotics as empirical treatment for community-acquired UTI [18]. In the present study, the most commonly used empirical antibiotics were carbapenems (39.7%), cephalosporins (33%), and piperacillin-tazobactam (13.4%). However, modifying treatment based on culture and antibiogram results is crucial in the management of ESBL-producing pathogens.

In older adults, UTIs often necessitate ICU admission due to immunosenescence and a high burden of comorbidities [19]. In our study, 25.6% of patients required ICU care, particularly those with chronic kidney disease, neurogenic bladder, and immunosuppression. CRP, procalcitonin, and SOFA scores were significantly elevated in this group. Similarly, risk prediction models for UTIs in the emergency department have identified the Charlson comorbidity index, leukocyte count, and procalcitonin levels as important predictors of ICU admission [20].

Although SOFA is a practical and widely used tool for early risk stratification, its relatively low sensitivity, particularly in elderly patients with atypical clinical presentations, should be carefully considered. In this population, SOFA may underestimate the severity of infection and therefore should not be used as a standalone prognostic tool. Instead, it should be interpreted in conjunction with clinical judgment, laboratory findings, and other severity indicators.

In this study, *E. coli* and *K. pneumoniae* isolated from urine cultures were associated with higher ICU admission and mortality rates, with resistant strains posing a greater risk, particularly MDR *Klebsiella* spp. Concurrent bacteremia was detected in 24.3% of patients, aligning with the reported prevalence rates of 9.8–42.7% in elderly UTI cases [4,20]. Immunosuppression, a high comorbidity burden, invasive procedures, and delayed diagnosis or treatment increase the risk of bacteremia. As expected, concurrent bacteremia worsened the clinical course, significantly increasing the need for ICU admission and mortality, highlighting the severity of the systemic inflammatory response.

Unlike previous studies primarily focusing on conventional clinical severity markers, our findings emphasize the substantial burden of multidrug-resistant (MDR) uropathogens in elderly patients. The high prevalence of MDR organisms and bacteremia observed in this multicenter cohort suggests that antimicrobial resistance plays a central role in determining clinical outcomes. These findings further emphasize the importance of considering antimicrobial resistance in early risk assessment and guiding empirical treatment strategies in elderly patients with UTIs. Additional stratified analyses showed that the effect of MDR on mortality was consistent across sex and catheter status, with no evidence of significant interaction. This suggests that these variables do not meaningfully modify the relationship between antimicrobial resistance and mortality in this population.

In this study, the in-hospital mortality rate was 14.6%, with 10.7% mortality on day 14 and 1.9% on day 28. The relatively higher rates observed in this study may be related to the inclusion of patients with greater comorbidity burden, prior healthcare exposure, and more severe clinical presentation. Reported mortality rates for urinary tract infections in elderly patients range from 2.2% to 33%, depending on demographic and clinical characteristics, access to care, infection severity, and pathogen resistance profiles [20,21,22]. These findings underscore the critical need for early diagnosis and timely treatment, particularly in elderly patients.

In the revised multivariable model, MDR/ESBL positivity, bacteremia, and SOFA score were independently associated with in-hospital mortality. Although CRP and procalcitonin were significantly associated with mortality in univariate analyses, they were not independent predictors in the multivariable model after adjustment for SOFA score, bacteremia, and other covariates. This finding suggests that their effects may be mediated through overall disease severity and the presence of bacteremia. Other mortality -associated factors reported in the literature include urinary catheter use, comorbidities, recent antibiotic exposure, and low serum albumin levels [10,21]. In our analysis, although catheter use was associated with mortality in univariate analysis, it was not an independent predictor after adjustment, suggesting that its effect may be mediated through infection severity and antimicrobial resistance. High CRP and procalcitonin levels reflect the severity of systemic inflammation and may serve as valuable biomarkers for assessing infection-related mortality risk [17].

These findings indicate that UTIs in older adult patients presenting to emergency departments are not only common but also associated with a high mortality risk. Early diagnosis and aggressive treatment are essential, especially in high-risk patients. To reduce mortality, greater focus on infection prevention, multidisciplinary management, and standardized protocols is needed, considering the rising antimicrobial resistance.

The strengths of this study include its large elderly cohort and multicenter design, which ensured a comprehensive dataset. However, several limitations should be acknowledged. First, the retrospective design may have introduced selection bias and potential data inaccuracies. In addition, only patients evaluated by infectious disease specialists were included, which may have resulted in the selection of more severe cases and may limit the generalizability of the findings. Furthermore, requiring microbiological confirmation for inclusion may have excluded clinically suspected UTI cases without documented culture growth. The inclusion of related urological conditions such as prostatitis may have introduced some degree of heterogeneity within the study population. Laboratory and microbiological variables presented in Table 4 were analyzed primarily using univariate methods; therefore, residual confounding cannot be excluded. An important limitation of this study is the lack of a direct measure of appropriate empirical antimicrobial therapy and time to treatment initiation. Due to the retrospective design, we used treatment modification according to culture results as a proxy variable; however, this may not fully capture the complexity of treatment appropriateness. Therefore, the potential mediating role of empirical therapy in the relationship between MDR and mortality should be interpreted with caution.

Although SOFA has been criticized for its limited sensitivity in some clinical settings, it remains a practical and widely used tool for early risk stratification in emergency departments.

Future research should adopt prospective, large-scale designs to better identify UTI-related mortality risk factors in the elderly and evaluate optimal treatment strategies, considering the increasing antimicrobial resistance of UTI pathogens.

Clinical Implications: The findings of this study highlight the need for a more individualized and risk-based approach in the management of elderly patients with UTIs. In particular, early recognition of patients who may require broader empirical antimicrobial coverage and closer clinical monitoring is essential in emergency settings. Integrating clinical scoring systems with microbiological risk factors may support more timely and appropriate therapeutic decisions, potentially improving patient outcomes.

## 5. Conclusions

This multicenter study demonstrates that urinary tract infections in elderly patients presenting to the emergency department carry a considerable risk of morbidity and mortality. Advanced age, comorbidity burden, and elevated inflammatory markers were strongly associated with poor outcomes. Resistant uropathogens, particularly MDR *Klebsiella* spp. and *E. coli*, markedly increased the likelihood of ICU admission and mortality. Early identification of high-risk patients through clinical evaluation, SOFA scoring, and timely laboratory assessment remains essential. Aligning empirical therapy with local resistance patterns and promptly initiating targeted treatment may help improve survival in this vulnerable population.

## Figures and Tables

**Table 1:** Baseline Characteristics of Patients According to ICU Admission and In-Hospital Mortality.

	Intensive Care Unit			Mortality		
Variables	Present (*N* = 301) *n* (%)	Absent (*N* = 874) *n* (%)	*p* Value	Present (*N* = 172) *n* (%)	Absent (*N* = 1003) *n* (%)	*p* Value
Gender						
Female	150 (49.8)	424 (48.5)	0.692 a	87 (50.6)	487 (48.6)	0.623 a
Male	151 (50.2)	450 (51.5)		85 (49.4)	516 (51.4)	
Age	78	74	<0.001 a	78	75	0.001
Antibiotic use (last 3 months)	180 (59.8)	356 (40.7)	<0.001 a	106 (61.6)	430 (42.9)	<0.001 a
Recurrent UTI (last 6 months)	120 (39.9)	299 (34.2)	0.077	69 (40.1)	350 (34.9)	0.187 a
Hospitalization (last 30 days)	112 (37.2)	239 (27.3)	0.001 a	73 (42.4)	278 (27.7)	<0.001 a
Nursing home	63 (20.9)	60 (6.9)	<0.001 a	37 (21.5)	86 (8.6)	<0.001 a
Prostatitis	9 (3)	3 (0.3)	<0.001 b	7 (4.1)	5 (0.5)	<0.001 a
Chronic kidney disease	63 (20.9)	115 (13.2)	0.001 a	36 (20.9)	142 (14.2)	0.022 a
Urolithiasis	61 (20.3)	116 (13.3)	0.003 a	36 (20.9)	141 (14.1)	0.020 a
Vesicoureteral reflux	2 (0.7)	2 (0.2)	0.272 b	0 (0)	4 (0.4)	1.0 b
Urinary malignancy	34 (9.3)	70 (7)	0.189 a	21 (8.7)	83 (7.4)	0.439 a
Immunosuppression	27 (9)	33 (3.8)	<0.001 a	20 (11.6)	40 (4.0)	<0.001 a
Non-urinary malignancy	36 (12)	38 (4.3)	<0.001 a	24 (14)	50 (5.0)	<0.001 a
Urinary intervention (last 3 months)	28 (9.3)	73 (8.4)	0.612 a	18 (10.5)	83 (8.3)	0.344 a
Urinary catheter	46 (15.3)	84 (9.6)	0.007 a	19 (11)	111 (11.1)	0.994 a
Nephrostomy catheter	14 (4.7)	57 (6.5)	0.240 a	9 (5.2)	62 (6.2)	0.629 a
Urinary stent	23 (7.6)	58 (6.6)	0.553 a	15 (8.7)	66 (6.6)	0.306 a
Neurogenic bladder	32 (10.6)	33 (3.8)	<0.001 a	17 (9.9)	48 (4.8)	0.007 a
Charlson comorbidity index	9.3 ± 11.4	7.9 ± 13.4	0.078 a	8.1 ± 4.9	8.3 ± 13.9	0.078 a

*a Pearson chi-square test, b Fisher’s exact test.*

**Table 2:** Univariate Analysis—In-Hospital Mortality.

Variables	In-Hospital Mortality −	In-Hospital Mortality +	OR (%95 GA)	*p*-Value
Age	75.00 (70.00–81.00)	78.00 (70.00–86.00)	1.04 (1.02–1.06)	<0.001
Male sex	87/581 (15.0%)	86/608 (14.1%)	0.94 (0.68–1.29)	0.747
Nursing home	136/1066 (12.8%)	37/123 (30.1%)	2.94 (1.92–4.50)	<0.001
Hospitalization (last 30 days)	98/827 (11.9%)	74/359 (20.6%)	1.93 (1.39–2.69)	<0.001
Antibiotic use (last 3 months)	65/640 (10.2%)	107/547 (19.6%)	2.15 (1.54–3.00)	<0.001
Recurrent UTI (last 6 months)	103/760 (13.6%)	70/429 (16.3%)	1.24 (0.89–1.73)	0.225
Healthcare-associated pyelonephritis	112/838 (13.4%)	61/351 (17.4%)	1.36 (0.97–1.92)	0.089
Diabetes mellitus	104/736 (14.1%)	69/453 (15.2%)	1.09 (0.79–1.52)	0.661
Chronic kidney disease	137/1009 (13.6%)	36/180 (20.0%)	1.59 (1.06–2.39)	0.033
Immunosuppression	153/1129 (13.6%)	20/60 (33.3%)	3.19 (1.82–5.60)	<0.001
Renal transplantation	167/1174 (14.2%)	6/15 (40.0%)	4.02 (1.41–11.44)	0.014
Urinary catheter	154/1057 (14.6%)	19/132 (14.4%)	0.99 (0.59–1.65)	1.000
Nephrostomy catheter	166/1120 (14.8%)	7/69 (10.1%)	0.65 (0.29–1.44)	0.372
Ureteral stent	157/1107 (14.2%)	16/82 (19.5%)	1.47 (0.83–2.60)	0.247
Any catheter (urinary catheter/nephrostomy/ureteral stent)	133/913 (14.6%)	40/276 (14.5%)	0.99 (0.68–1.46)	1.000
Urinary intervention within the last 3 months	154/1087 (14.2%)	19/102 (18.6%)	1.39 (0.82–2.35)	0.283
Urolithiasis	163/1132 (14.4%)	10/57 (17.5%)	1.26 (0.63–2.55)	0.642
Benign prostatic hyperplasia	154/1017 (15.1%)	19/172 (11.0%)	0.70 (0.42–1.16)	0.196
Prostatitis	166/1177 (14.1%)	7/12 (58.3%)	8.53 (2.67–27.18)	<0.001
Neurogenic bladder	156/1122 (13.9%)	17/67 (25.4%)	2.11 (1.18–3.74)	0.016
Any malignancy	134/1027 (13.0%)	39/162 (24.1%)	2.11 (1.41–3.16)	<0.001
Altered mental status	93/829 (11.2%)	80/360 (22.2%)	2.26 (1.63–3.14)	<0.001
Fever	57/409 (13.9%)	116/780 (14.9%)	1.08 (0.77–1.52)	0.728
WBC > 11,000/µL	56/424 (13.2%)	117/764 (15.3%)	1.19 (0.84–1.67)	0.368
C-reactive protein (mg/L)	112.00 (67.00–180.25)	142.00 (78.00–239.00)	1.00 (1.00–1.00)	<0.001
Procalcitonin (µg/mL)	1.63 (0.27–10.60)	2.90 (0.54–9.35)	1.01 (1.00–1.02)	0.012
Creatinine (mg/dL)	1.00 (0.70–1.67)	1.75 (0.87–3.60)	1.01 (1.00–1.02)	<0.001
Urea (mg/dL)	41.00 (26.00–63.00)	58.00 (31.00–109.00)	1.01 (1.01–1.01)	<0.001
GFR ≤ 50 mL/min/1.73 m^2^	74/715 (10.3%)	99/474 (20.9%)	2.29 (1.65–3.17)	<0.001
Lactate (mmol/L)	1.56 (1.00–2.40)	2.74 (1.52–4.70)	1.44 (1.33–1.56)	<0.001
Charlson comorbidity index	5.00 (4.00–7.00)	7.00 (5.00–10.00)	1.00 (0.99–1.01)	<0.001
Pitt bacteremia score	1.00 (0.00–1.00)	4.00 (3.50–6.00)	2.19 (1.83–2.62)	<0.001
SOFA score	2.00 (0.00–4.00)	8.00 (4.00–10.00)	1.52 (1.44–1.62)	<0.001
MDR	107/878 (12.2%)	59/150 (39.3%)	4.67 (3.18–6.87)	<0.001
ESBL or MDR (broad definition)	34/358 (9.5%)	132/670 (19.7%)	2.34 (1.57–3.49)	<0.001
Bacteremia (positive blood culture)	81/873 (9.3%)	92/295 (31.2%)	4.43 (3.17–6.20)	<0.001
Treatment modification	75/585 (12.8%)	98/602 (16.3%)	1.32 (0.96–1.83)	0.108

*Continuous variables are presented as median (Q1–Q3); categorical variables as n (%). Mann–Whitney U, Fisher’s exact, and chi-square (χ^2^) tests were used.*

**Table 3:** Univariate Analysis—ICU Admission Requirement.

Variables	ICU Admission −	ICU Admission +	OR (%95 GA)	*p*-Value
Age	74.00 (70.00–81.00)	78.00 (70.75–84.00)	1.04 (1.02–1.06)	<0.001
Male sex	151/581 (26.0%)	153/608 (25.2%)	0.96 (0.74–1.24)	0.795
Nursing home residence	241/1066 (22.6%)	63/123 (51.2%)	3.59 (2.45–5.26)	<0.001
Hospitalization within the last 30 days	188/827 (22.7%)	115/359 (32.0%)	1.60 (1.22–2.11)	<0.001
Antibiotic use within the last 3 months	120/640 (18.8%)	183/547 (33.5%)	2.18 (1.67–2.84)	<0.001
History of UTI within the last 6 months	182/760 (23.9%)	122/429 (28.4%)	1.26 (0.97–1.65)	0.102
Healthcare-associated pyelonephritis	208/838 (24.8%)	96/351 (27.4%)	1.14 (0.86–1.51)	0.401
Diabetes mellitus	177/736 (24.0%)	127/453 (28.0%)	1.23 (0.94–1.60)	0.144
Chronic kidney disease	241/1009 (23.9%)	63/180 (35.0%)	1.72 (1.22–2.41)	0.002
Immunosuppression	277/1129 (24.5%)	27/60 (45.0%)	2.52 (1.49–4.26)	<0.001
Renal transplantation	298/1174 (25.4%)	6/15 (40.0%)	1.96 (0.69–5.55)	0.321
Urinary catheter	258/1057 (24.4%)	46/132 (34.8%)	1.66 (1.13–2.43)	0.013
Nephrostomy catheter	292/1120 (26.1%)	12/69 (17.4%)	0.60 (0.32–1.13)	0.144
Ureteral stent	280/1107 (25.3%)	24/82 (29.3%)	1.22 (0.75–2.00)	0.506
Any catheter (urinary catheter/nephrostomy/ureteral stent)	225/913 (24.6%)	79/276 (28.6%)	1.23 (0.91–1.66)	0.212
Urinary intervention within the last 3 months	275/1087 (25.3%)	29/102 (28.4%)	1.17 (0.75–1.84)	0.566
Urolithiasis	288/1132 (25.4%)	16/57 (28.1%)	1.14 (0.63–2.07)	0.773
Benign prostatic hyperplasia	271/1017 (26.6%)	33/172 (19.2%)	0.65 (0.44–0.98)	0.048
Prostatitis	295/1177 (25.1%)	9/12 (75.0%)	8.97 (2.41–33.35)	<0.001
Neurogenic bladder	272/1122 (24.2%)	32/67 (47.8%)	2.86 (1.74–4.70)	<0.001
Any malignancy	239/1027 (23.3%)	65/162 (40.1%)	2.21 (1.56–3.12)	<0.001
Altered mental status	161/829 (19.4%)	143/360 (39.7%)	2.73 (2.08–3.59)	<0.001
Fever	114/409 (27.9%)	190/780 (24.4%)	0.83 (0.64–1.09)	0.212
WBC > 11,000/µL	113/424 (26.7%)	190/764 (24.9%)	0.91 (0.69–1.19)	0.545
C-reactive protein (mg/L)	112.00 (68.00–177.00)	127.50 (70.53–228.00)	1.00 (1.00–1.00)	0.009
Procalcitonin (µg/mL)	1.90 (0.30–10.70)	1.53 (0.35–6.42)	1.01 (1.00–1.02)	0.948
Creatinine (mg/dL)	0.99 (0.70–1.60)	1.60 (0.90–3.10)	1.02 (1.01–1.03)	<0.001
Urea (mg/dL)	40.10 (26.00–59.00)	56.00 (30.00–102.00)	1.01 (1.01–1.01)	<0.001
GFR ≤ 50 mL/min/1.73 m^2^	134/715 (18.7%)	170/474 (35.9%)	2.42 (1.86–3.16)	<0.001
Lactate (mmol/L)	1.50 (1.00–2.30)	2.36 (1.44–4.50)	1.58 (1.44–1.73)	<0.001
Charlson comorbidity index	5.00 (4.00–6.00)	7.00 (5.00–10.00)	1.01 (1.00–1.02)	<0.001
Pitt bacteremia score	1.00 (0.00–1.00)	4.00 (3.00–6.00)	2.65 (2.13–3.29)	<0.001
SOFA score	1.00 (0.00–4.00)	6.00 (3.00–9.00)	1.57 (1.48–1.66)	<0.001
MDR	215/878 (24.5%)	79/150 (52.7%)	3.43 (2.40–4.90)	<0.001
ESBL or MDR (broad definition)	74/358 (20.7%)	220/670 (32.8%)	1.88 (1.39–2.54)	<0.001
Bacteremia (positive blood culture)	177/873 (20.3%)	124/295 (42.0%)	2.85 (2.15–3.79)	<0.001
Treatment modification	141/585 (24.1%)	163/602 (27.1%)	1.17 (0.90–1.52)	0.268

*Continuous variables are presented as median (Q1–Q3); categorical variables as n (%). Mann–Whitney U, Fisher’s exact, and chi-square (χ^2^) tests were used.*

**Table 4:** Multivariable Logistic Regression Analysis for In-Hospital Mortality.

Variable	Beta	Adjusted OR (95% CI)	*p*-Value
MDR/ESBL positivity	0.735	2.09 (1.24–3.50)	0.005
Bacteremia	0.741	2.10 (1.32–3.35)	0.002
SOFA score	0.425	1.53 (1.42–1.65)	<0.001
log(CRP + 1)	0.070	1.07 (0.83–1.38)	0.588
log(Procalcitonin + 1)	0.004	1.00 (0.82–1.23)	0.966
Any catheter	−0.321	0.73 (0.43–1.23)	0.232
Charlson comorbidity index	0.016	1.02 (0.99–1.04)	0.153
Age	0.027	1.03 (1.00–1.06)	0.044
Mental status changes	0.483	1.62 (1.03–2.54)	0.035
GFR ≤ 50	−0.796	0.45 (0.27–0.75)	0.002
Treatment modification	0.222	1.25 (0.80–1.95)	0.326

***Model performance:** AUC = 0.858 (95% CI: 0.819–0.897); Hosmer–Leme show goodness-of-fit test: χ^2^ = 10.12, df = 8, p = 0.257.*

**Table 5:** Multivariable Logistic Regression Analysis for 28-Day Mortality (Sensitivity Analysis).

Variable	Beta	Adjusted OR (95% CI)	*p*-Value
MDR/ESBL positivity	0.637	1.89 (1.12–3.19)	0.017
Bacteremia	0.615	1.85 (1.15–2.97)	0.011
SOFA score	0.373	1.45 (1.35–1.56)	<0.001
log(CRP + 1)	−0.002	1.00 (0.78–1.28)	0.987
log(Procalcitonin + 1)	−0.005	0.99 (0.81–1.22)	0.958
Any catheter	−0.204	0.82 (0.48–1.37)	0.443
Charlson comorbidity index	0.015	1.02 (0.99–1.04)	0.181
Age	0.017	1.02 (0.99–1.04)	0.219
Mental status changes	0.637	1.89 (1.21–2.96)	0.005
GFR ≤ 50	−0.781	0.46 (0.27–0.77)	0.003
Treatment modification	0.176	1.19 (0.76–1.86)	0.437

*Model performance: AUC = 0.835 (95% CI: 0.791–0.878); Hosmer–Leme show goodness-of-fit test: p = 0.624.*

## Data Availability

All data needed to support the conclusions are present in the paper. Raw data are available from the corresponding author, P.Y.A., upon reasonable request.

## Supplementary Materials

The following supporting information can be downloaded at: https://www.mdpi.com/article/10.3390/diagnostics16111708/s1, Table S1: Multivariable Logistic Regression Analysis Stratified by Sex; Table S2: Multivariable Logistic Regression Analysis Stratified by Catheter Status; Table S3 (Table 2-bis): Univariate Analysis—28-Day Mortality (Sensitivity Analysis); Figure S1: ROC Curves for Three Outcomes.

## Abbreviations

The following abbreviations are used in this manuscript:

BUN	Blood urea nitrogen
CFU/mL	Colony-forming units per milliliter
CRP	C-reactive protein
*E. coli*	*Escherichia coli*
ESBL	Extended-spectrum beta-lactamase
GFR	Glomerular filtration rate
ICU	Intensive care unit
*K. pneumoniae*	*Klebsiella pneumoniae*
MDR	Multidrug-resistant
SOFA	Sequential Organ Failure Assessment
UTI	Urinary tract infection
UTIs	Urinary tract infections
