# Supplementary material for: Impact of Multidrug-Resistant Uropathogens on Mortality in Elderly Patients with Urinary Tract Infections: A Multicenter Retrospective Study
Source: Diagnostics (Basel). 2026 Jun 2;16(11):1708. doi: 10.3390/diagnostics16111708 (PMC13256847; doi:10.3390/diagnostics16111708)
Supplement: Supplementary file 1 [file diagnostics-16-01708-s001.zip › diagnostics-4266949-supplementary.pdf]

Supplementary Analyses in Response to Reviewer Comments — Parallel Outcome Results

Table S1. Multivariable Logistic Regression Analysis Stratified by Sex

Separate multivariable logistic regression models were constructed for female and male patients for the in-hospital mortality outcome.

Strata	(n, included in model)	Number of events	MDR/ESBL OR (%95 GA)	p-value	AUC (95% CI)
**Female**	423	77	1.93 (0.92–4.08)	0.083	0.878 (0.826–0.929)
**Male**	410	76	2.24 (1.06–4.73)	0.034	0.857 (0.802–0.912)

MDR × Sex interaction test: OR = 1.02 (95% CI: 0.36–2.87), p = 0.965, indicating no statistically significant difference in the effect of MDR on in-hospital mortality between sexes.

Table S2. Multivariable Logistic Regression Analysis Stratified by Catheter Status

Separate multivariable logistic regression models were constructed for patients with and without catheterization for the in-hospital mortality outcome.

Strata	(n, included in model)	Number of events	MDR/ESBL OR (%95 GA)	p-value	AUC (95% CI)
**No catheter**	633	118	2.20 (1.23–3.93)	0.008	0.867 (0.824–0.910)
**Catheter present**	200	35	2.11 (0.62–7.23)	0.235	0.826 (0.738–0.914)

MDR × Catheter interaction test: OR = 0.88 (95% CI: 0.23–3.45), p = 0.858, indicating no statistically significant difference in the effect of MDR on in-hospital mortality according to catheter status.

Key finding:

Sex and catheter status do not act as effect modifiers of the association between MDR and mortality. Point estimates are consistent across all strata. Therefore, retaining these variables as covariates in the primary model is a statistically efficient approach.

Table S3 (Table 2-bis). Univariate Analysis — 28-Day Mortality (Sensitivity Analysis)

Variable	28-day mortality -	28-day mortality +	OR (%95 GA)	p-value
**Age**	75.00 (70.00–81.00)	78.00 (70.00–85.00)	1.03 (1.01–1.06)	0.006
**Male sex**	79/580 (13.6%)	74/605 (12.2%)	0.88 (0.63–1.24)	0.531
**Nursing home residence**	121/1062 (11.4%)	32/123 (26.0%)	2.73 (1.75–4.27)	<0.001

**Hospitalization within the last 30 days**	86/824 (10.4%)	67/358 (18.7%)	1.98 (1.40–2.80)	<0.001
**Antibiotic use within the last 3 months**	57/637 (8.9%)	96/546 (17.6%)	2.17 (1.53–3.08)	<0.001
**History of UTI within the last 6 months**	91/756 (12.0%)	62/429 (14.5%)	1.23 (0.87–1.75)	0.271
**Healthcare-associated pyelonephritis**	98/835 (11.7%)	55/350 (15.7%)	1.40 (0.98–2.00)	0.077
**Diabetes mellitus**	90/732 (12.3%)	63/453 (13.9%)	1.15 (0.82–1.63)	0.475
**Chronic kidney disease**	121/1006 (12.0%)	32/179 (17.9%)	1.59 (1.04–2.44)	0.042
**Immunosuppression**	137/1125 (12.2%)	16/60 (26.7%)	2.62 (1.44–4.78)	0.002
**Renal transplantation**	148/1170 (12.6%)	5/15 (33.3%)	3.45 (1.16–10.24)	0.047
**Urinary catheter**	136/1053 (12.9%)	17/132 (12.9%)	1.00 (0.58–1.71)	1.000
**Nephrostomy catheter**	148/1116 (13.3%)	5/69 (7.2%)	0.51 (0.20–1.29)	0.207
**Ureteral stent**	137/1103 (12.4%)	16/82 (19.5%)	1.71 (0.96–3.04)	0.094
**Any catheter (urinary catheter/nephrostomy/ureteral stent)**	116/909 (12.8%)	37/276 (13.4%)	1.06 (0.71–1.57)	0.859
**Urinary intervention within the last 3 months**	137/1083 (12.7%)	16/102 (15.7%)	1.28 (0.73–2.26)	0.472
**Urolithiasis**	143/1128 (12.7%)	10/57 (17.5%)	1.47 (0.72–2.97)	0.386
**Benign prostatic hyperplasia**	134/1013 (13.2%)	19/172 (11.0%)	0.81 (0.49–1.36)	0.505
**Prostatitis**	146/1173 (12.4%)	7/12 (58.3%)	9.85 (3.09–31.44)	<0.001
**Neurogenic bladder**	141/1118 (12.6%)	12/67 (17.9%)	1.51 (0.79–2.89)	0.285
**Any malignancy**	117/1024 (11.4%)	36/161 (22.4%)	2.23 (1.47–3.39)	<0.001
**Altered mental status**	80/827 (9.7%)	73/358 (20.4%)	2.39 (1.69–3.38)	<0.001
**Fever**	48/407 (11.8%)	105/778 (13.5%)	1.17 (0.81–1.68)	0.460
**WBC > 11,000/µL**	50/423 (11.8%)	103/761 (13.5%)	1.17 (0.81–1.68)	0.452
**C-reactive protein (mg/L)**	113.44 (68.00–183.00)	134.72 (76.00–202.00)	1.00 (1.00–1.00)	0.011
**Procalcitonin (µg/mL)**	1.66 (0.28–10.60)	2.90 (0.52–9.30)	1.01 (1.00–1.02)	0.033
**Creatinine (mg/dL)**	1.00 (0.70–1.67)	1.63 (0.80–3.54)	1.01 (1.00–1.02)	<0.001

**Urea (mg/dL)**	41.00 (26.00–63.20)	58.00 (32.00–102.00)	1.01 (1.00–1.01)	<0.001
**GFR ≤ 50 mL/min/1.73 m²**	69/714 (9.7%)	84/471 (17.8%)	2.03 (1.44–2.86)	<0.001
**Lactate (mmol/L)**	1.60 (1.00–2.47)	2.75 (1.52–4.56)	1.36 (1.26–1.47)	<0.001
**Charlson comorbidity index**	5.00 (4.00–7.00)	7.00 (5.00–10.00)	1.00 (0.99–1.01)	<0.001
**Pitt bacteremia score**	1.00 (0.00–2.00)	4.00 (3.25–6.75)	1.90 (1.63–2.23)	<0.001
**SOFA score**	2.00 (0.00–4.00)	8.00 (3.00–10.00)	1.46 (1.38–1.54)	<0.001
**MDR**	97/874 (11.1%)	49/150 (32.7%)	3.89 (2.60–5.80)	<0.001
**ESBL or MDR (broad definition)**	30/356 (8.4%)	116/668 (17.4%)	2.28 (1.49–3.49)	<0.001
**Bacteremia (positive blood culture)**	74/871 (8.5%)	79/293 (27.0%)	3.98 (2.80–5.65)	<0.001
**Treatment modification**	67/583 (11.5%)	86/600 (14.3%)	1.29 (0.92–1.81)	0.171

Figure S1. ROC Curves for Three Outcomes

Discriminative performance of the multivariable models for in-hospital mortality (primary outcome), 28-day mortality (sensitivity analysis), and ICU admission.

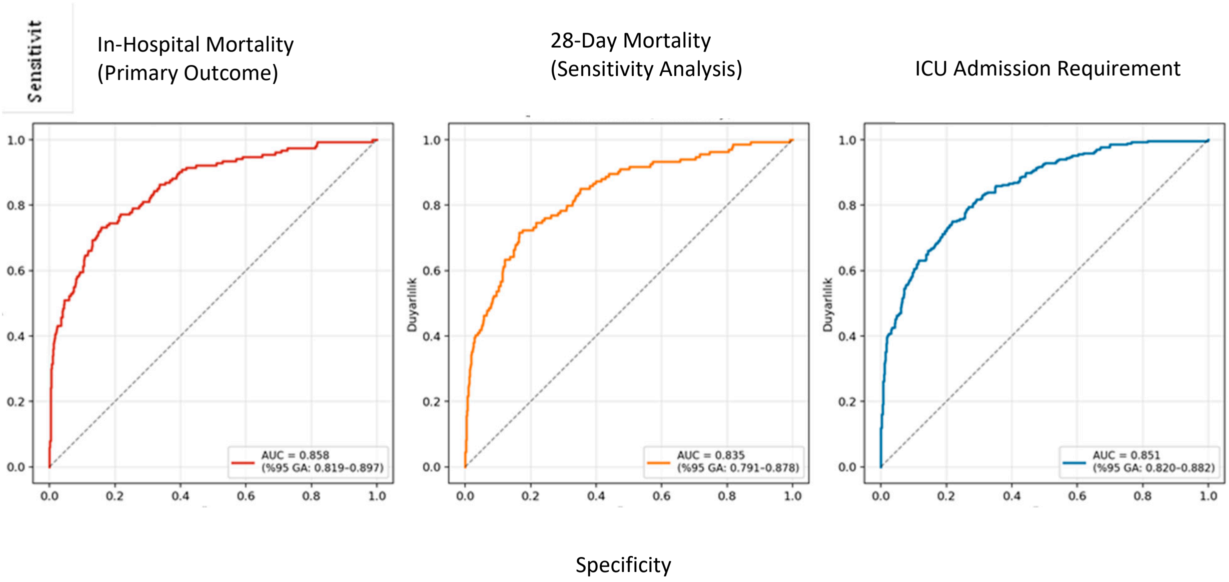

Sensitivit
In-Hospital Mortality
(Primary Outcome)
28-Day Mortality
(Sensitivity Analysis)
ICU Admission Requirement
Duyarlılık
AUC = 0.858
(%95 GA: 0.819–0.897)
AUC = 0.835
(%95 GA: 0.791–0.878)
AUC = 0.851
(%95 GA: 0.820–0.882)
0.0
0.2
0.4
0.6
0.8
1.0
Specificity
